# Abdominal venous thrombosis presenting in myeloproliferative neoplasm with *JAK2 V617F *mutation: a case report

**DOI:** 10.1186/1752-1947-6-102

**Published:** 2012-04-05

**Authors:** Naveen Pemmaraju, James Peter Hamilton, Andrew M Cameron, Stephen Sisson, Alison R Moliterno

**Affiliations:** 1 MD Anderson Cancer Center, 1515 Holcombe Boulevard, Houston, TX 77030, USA; 2 Johns Hopkins Hospital, 600 North Wolfe Street, Baltimore, MD 21287, USA

## Abstract

**Introduction:**

An unprovoked thombotic event in a patient is cause for further evaluation of an underlying hypercoaguable state. The investigation should include a thorough search, including checking for a variety of known inherited and acquired hypercoaguble states (protein C or S deficiency, anti-phospholipid antibodies, and anti-thrombin III deficiency) and gene mutations that predispose patients to an increased risk of clotting (for example, prothrombin gene 20210 mutation, factor V Leiden, and the *JAK2 V617F *mutation).

**Case presentation:**

We report the case of a 38-year-old Caucasian woman with spontaneous, unprovoked abdominal venous thrombosis and demonstrate how testing for the *JAK2 V617F *mutation was useful in unmasking an underlying hypercoaguable state.

**Conclusions:**

*JAK2 V617F*-positive myeloproliferative neoplasm was diagnosed. This case illustrates the importance of testing for *JAK2 V617F *in patients presenting with Budd-Chiari syndrome, even in the absence of overt hematologic abnormalities, in order to establish a diagnosis of underlying myeloproliferative neoplasm.

## Introduction

An unprovoked thombotic event in a patient is cause for further evaluation of an underlying hypercoaguable state. The investigation should include a thorough search, including checking for a variety of known inherited and acquired hypercoaguble states and gene mutations that predispose patients to an increased risk of clotting. Therefore, the differential diagnosis of underlying etiologies of Budd-Chiari syndrome (BCS) includes the following: myeloproliferative neoplasm (MPN), malignancy, paroxysmal nocturnal hemoglobinuria, anti-phospholipid syndrome, sickle cell anemia, and inherited hypercoaguable states, including factor V Leiden, prothrombin 20210 mutation, and protein C and S and anti-thrombin deficiencies [1].

The MPNs are a related group of diseases that have a common origin of an acquired stem cell or progenitor cell defect that leads to the development of overproliferation of the myeloid series, manifest as essential thrombocytosis (ET), polycythemia vera (PV), or primary myelofibrosis (PMF). In 2005, it was demonstrated that MPNs are connected by an acquired somatic mutation of the JAK2 gene, *JAK2 V617F *[2], which is found in approximately 95% of patients with PV and in 50% of patients with ET or PMF.

## Case presentation

A 38-year-old Caucasian woman presented to our hospital with two months of epigastric abdominal pain associated with bloating. She denied nausea or vomiting, history of acid reflux, constipation, or diarrhea. She had no known trauma. She affirmed complaints of fatigue and decreased appetite but denied fevers, chills, night sweats, or weight loss. She denied a history of liver or blood disorders.

The initial evaluation revealed a jaundiced young woman with abdominal ascites and trace lower-extremity edema. Laboratory studies revealed elevated levels of gamma glutamyl transferase (GGT), total bilirubin, and CA-125. A computed tomography (CT) scan with oral and intravenous contrast of the abdomen and pelvis was performed, demonstrating a heterogeneous appearance of the liver and caudate lobe hypertrophy, ascites, and a cystic mass in the right ovary. The liver abnormalities described were thought to be consistent with diffuse metastatic involvement of the liver, and the ovaries were thought to be a possible primary source. Our patient then underwent laparoscopic oopherectomy and liver biopsy to evaluate for underlying malignancy. No evidence of either ovarian or hepatic malignancy was found, but the liver biopsy revealed fibrous obliteration of the central hepatic veins and patchy areas of sinusoidal dilatation diagnostic of hepatic vein obstruction, or BCS. A triphasic CT scan of the liver with three-dimensional reconstruction confirmed occlusion of the hepatic veins and associated ascites, splenomegaly, and abdominal varices.

Our patient's medical history was notable for one episode of unexplained syncope one year prior and migraine with aura. Medications included warfarin and aspirin. Our patient was married and lived with her husband. She worked as a busy executive, frequently taking international flights. She had never smoked and she drank one or two glasses of wine per week. The only family history of a thrombosis was in a paternal aunt with a deep vein thrombosis in the setting of a long car ride. Our patient had no family history of cancer or other blood disorders.

Laboratory findings revealed a white blood cell count of 3640 K/μL, hemoglobin of 11.1 g/dL, mean corpuscular volume (MCV) of 80fL, and a platelet count of 252,000 K/μL. Erythropoietin level was 22 mIU/mL (normal is 4.1 to 19.5), international normalized ratio was 1.4 on warfarin therapy, ammonia 45 Umol/L (10 to 47 Umol/L), total bilirubin 3.1 mg/dL, direct bilirubin 0.6, albumin 3.7 g/dL, and alkaline phosphate 158 IU/L. Alanine aminotransferase and aspartate aminotransferase were normal. GGT was elevated to 120 U/L (12 to 48 U/L). CA-125 was 55 U/mL (normal is less than 35 U/mL). Liver biopsy results showed central hepatic veins with pericentral fibrosis and mild portal fibrosis and patchy areas of moderate sinusoidal dilatation consistent with chronic BCS.

Our patient was evaluated for a hypercoaguable state, including tests for lupus anticoagulant and anti-phospholipid antibodies, factor V Leiden, and prothombin 20210 mutation. The results of these tests were negative. Levels of anti-thrombin and protein C and S were normal. The result of one test was found from the hypercoaguable workup to be positive: *JAK2 V617F *mutation with an allele percentage of 36%. Complete blood counts obtained in the five years prior to her diagnosis were notable for thrombocytosis (Table 1).

**Table 1:** Complete blood counts in a patient with *JAK2 V617F*-associated Budd-Chiari syndrome

Date	Hemoglobin, g/dL	WBCs, K/μL	Platelets, K/μL	Clinical events
Dec. 9, 2002	11.3	4.1	349	
Dec. 19, 2002	12.9	6.6	416	
Jan. 10, 2006	13.8	8.6	522	
Dec. 27, 2007	13.9	6.9	354	Symptoms of BCS
Jan. 7, 2008	12.5	5.5	379	Post-oophorectomy
March 25, 2008	13.5	4.7	261	Post-TIPS
April 29, 2008	11.1	3.6	252	Follow-up visit
July 29, 2008	12.5	3.5	239	Follow-up visit
Sept. 15, 2009	13.2	4.7	287	Follow-up visit
May 4, 2010	14.1	5.4	293	Follow-up visit
June 3, 2011	13.3	5.2	285	Follow-up visit

Normal ranges of hemoglobin, white blood cells (WBCs), and platelets are 12 to 15 g/dL, 4.5 to 11 K/μL, and 150 to 450 K/μL, respectively. BCS, Budd-Chiari syndrome; TIPS, transjugular intrahepatic porto-systemic shunt.

## Discussion

On the basis of the new-onset BCS combined with the finding of the *JAK2 V617F *mutation, our patient's condition was diagnosed as an underlying chronic MPN. Thromboses, including abdominal vein thromboses, constitute a major etiology of morbidity and mortality in MPN [3]. MPN now constitutes the most common cause of abdominal venous thromboses, accounting for approximately 50% of BCS cases and 25% of portal vein thromboses [4]. A recent study demonstrated MPN to be the most prevalent underlying condition (49%) in a series of 163 incident cases of BCS [1]. In most of these patients, portal hypertension was a typical feature. This becomes important because it may help to explain the surprisingly low or normal blood counts observed in BCS *JAK2 V617F*-positive patients; the subsequent hemodilution and hypersplenism resulting from the portal hypertension may decrease the actual hemoglobin and other blood cell counts, making the diagnosis of MPN quite challenging in many cases [4]. Masking of the MPN by subsequent hemodilution from portal hypertension or even from hepatic dysfunction is evident in our patient, in whom the platelet count normalized from the previously mild elevation evident in the years before her BCS diagnosis (Table 1). Several groups have hypothesized that endothelial cell (EC) dysfunction may contribute to the prothrombotic state in MPN. In a seminal study, Sozer and colleagues [5] demonstrated that the *JAK2 V617F *mutation was present in the ECs from venules of liver biopsy specimens obtained from two patients with BCS and PV, suggesting that ECs in PV are involved in the malignant process and contribute to the prothrombotic state found in this disorder.

It has been suspected for decades that primary, occult MPN may play a role in the prevalence of BCS, especially in young women. One study demonstrated that erythroid colony formation in the absence of erythropoietin, a reliable indicator of MPN in the pre-*JAK2 V617F *era, was present in 16 out of 20 patients with BCS, most of whom were young women (18 to 45 years old) [6]. The authors concluded, some 20 years prior to the elucidation of the *JAK2 V617F *mutation, that primary MPN is a major cause of BCS in young women. Many cases labeled as idiopathic BCS have been subsequently found to be secondary to underlying myeloproliferative disease upon *JAK2 V617F *testing [7]. In the literature reported to date, the largest series relating *JAK2 V617F *mutation status to abdominal venous thrombosis showed that *JAK2 V617F*-associated BCS was predominantly a disease of young women, that it was associated with relatively low *JAK2 V617F *allele burdens, and that the majority of the patients did not have additional identified hypercoagulable risk factors, all features common to our patient [4]. Also, the most common risks for thrombotic disease in MPN are older age (> 60 years), elevated white cell count, and prior thrombotic events [8], none of which applies to either our patient or the MPN BCS patients at large [4]. The discrepancy between the absence of traditional MPN thrombotic risk factors and the low *JAK2 V617F *allele burdens in the patients with BCS indicates that other important risk factors for this devastating presentation, particularly relevant to young women with MPN, remain undefined [9]. It is interesting to note that our patient's JAK2 V617 allele burden at diagnosis measured only 36%. *JAK2 V617F *allele burdens are lower in women in comparison with men, suggesting that gender is an important modifier of disease phenotype and may in part account for differences in disease presentation and complications between men and women with *JAK2 V617F*-positive MPNs [3,10].

It is important to note that, because *JAK2 V617F *is present in 95% of patients with PV but only in approximately 50% of patients with ET or PMF, a negative *JAK2 V617F *study should not by itself preclude the diagnosis of an underlying MPN. In such cases, further investigation, including a bone marrow biopsy, should be sought as part of a complete workup.

## Conclusions

Our patient's condition was diagnosed as *JAK2 V617F*-positive MPN, most consistent with ET (Table 1). She subsequently underwent a transjugular intrahepatic porto-systemic shunt (TIPS) placement to decompress her hepatic obstruction and this was followed by indefinite warfarin-based anticoagulation. Her clinical status and blood counts have been stable in the three years since her TIPS procedure (Table 1), and her hepatic function remains excellent. This case illustrates the importance of testing for *JAK2 V617F *in patients presenting with BCS, even in the absence of overt hematologic abnormalities, in order to establish a diagnosis of underlying MPN. Although molecular testing with JAK2 is rather specific to the MPN classification, it alone cannot specify which MPN the patient harbors [11,12]. Therefore, further studies, including red cell mass, regular complete blood cell counts, and assessment of the *JAK2 V617F *allele percentage over time, may be indicated to further aid in the individual MPN diagnosis of our patient [10]. It is important to recognize that CA-125 is not a reliable screening test for the diagnosis of ovarian cancer in pre-menopausal woman. In addition, as in this case, CA-125 can be elevated in the presence of liver disease and can be a sensitive marker of ascites [13]. Finally, non-invasive imaging is of critical importance in the diagnostic evaluation of abdominal vein thrombosis, and the majority of cases are identified by Doppler ultrasound. In some instances, high-quality multi-phasic examinations of the liver are often needed to confirm the diagnosis of BCS.

## Abbreviations

BCS: Budd-Chiari syndrome; CT: computed tomography; EC: endothelial cell; ET: essential thrombocytosis; GGT: gamma glutamyl transferase; MPN: myeloproliferative neoplasm; PMF: primary myelofibrosis; PV: polycythemia vera; TIPS: transjugular intrahepatic porto-systemic shunt.

## Consent

Written informed consent was obtained from the patient for publication of this manuscript and accompanying images. A copy of the written consent is available for review by the Editor-in-Chief of this journal.

## Competing interests

The authors declare that they have no competing interests.

## Authors' contributions

NP and ARM helped to design and write the manuscript and to analyze and interpret the hematologic and internal medicine aspects of the patient data from this case. SS helped to analyze and interpret the hematologic and internal medicine aspects of the patient data from this case. JPH and AMC analyzed and assisted with the gastrointestinal and hepatologic interpretation of data from this case. All authors read and approved the final manuscript.
